# 

**Published:** 1895-09-07

**Authors:** 


**Notices of Births, Deaths, and Marriages** are inserted in this column at a charge of 2s. 6d. for an announcement not exceeding four lines.

**Notice to Advertisers and Subscribers.**

All Advertisements, Orders for Copies of The Hospital and Business Communications should be addressed to **The Manager, NOT TO THE EDITOR**, The Hospital, 428, Strand, London, W.C.

The Cost of Subscription, post free, is as follows:—Per week, 2½d.; per quarter, 2s. 8d.; per half-year, 5s. 3d.; per annum, 10s. 6d. Foreign:—Per Annum, 15s. 2d.; payable, in all cases, in advance.

**NOTICE TO CORRESPONDENTS.**

All MS., Letters, Books for Review, and other matters intended for the Editor should be addressed The Editor, The Lodge, Porchester Square, London, W.

The Editor cannot undertake to return rejected MS., even when accompanied by stamped directed envelope.

**"Burdett's Hospital Annual," 1895,**

Sixth year of publication. 950 pp. Scarlet cloth, gilt lettered. Price 5s. A most complete guide to British, Colonial, Indian, and American Hospitals, Dispensaries, Nursing and Convalescent Institutions, and Asylums. A few copies of the "Annual" for 1894 may still be obtained on application to the Publishers, The Scientific Press (Limited), 428, Strand, London, W.C.

**NOTICE.—Vol. XVII. of "The Hospital" (October, 1894, to April, 1895), in handsome cloth binding, is on sale at the Office of this Journal. Price 6s. Cases for binding the Numbers contained in this Volume, 1s. 6d. Index to Vol. XVII., 2½d., post free.**

**The Monthly Part for September is now ready, price 6d.; by post, 8d.**

### SCRAPS AND GLEANINGS.

THE opening of the new infirmary at Southport will not take place until September 27th.

THE *Assistance Publique* has received a bequest of £72,000 from M. Rouchard, a late member of the Conseil de Surveillance.

MR. PASSMORE EDWARDS has offered to erect a building, if a site is provided, for a free library in the parish of St. George's, Southwark.

THE progress which Science has made in the Bordeaux hospitals was enthusiastically commented on at the recent Gynæcological Congress held in that city.

IN thirty-three of the largest English towns the births registered during the week ending August 10th numbered 5,816, whilst 4,225 deaths were recorded in the same period.

THE thirty persons recently poisoned by potted meat at Wakefield are, fortunately, all likely to recover. The unsold portion of the supply was seized by the police for analysis.

SERIOUS damage to museum specimens, diagrams, and microscopic preparations has resulted from fire in one of the buildings of the Edinburgh Extra Mural School of Medicine.

MR. ROSE had a number of cases before him at the West London Police Court the other day, in which summonses had been issued against parents for failing to have their children vaccinated.

THE census of metropolitan paupers, taken during the last week in July, showed that 98,354 persons were in receipt of relief; in the corresponding week of last year the return gave 93,764 paupers.

THE Local Government Board has refused to sanction the scheme by which certain members of the Poplar Board of Guardians proposed to establish at Dunton, Essex, a farm colony for the unemployed.

DURING the military manœuvres in the New Forest, the great heat which prevailed on the day of the march past caused so many men to drop out of the ranks that the ambulance wagons were quite filled.

A PATIENT admitted to Bethnal Green Infirmary suffering from delirium tremens is stated to have committed suicide in the padded room by hanging himself with a sheet which he had torn up and used as a cord.

THE museum and library at the Sanitary Institute will be opened free during the months of September, October, and November to students attending the course of lectures for sanitary officers commencing on September 3rd.

OVER £130 was taken last week as the outcome of the bazaar held aboard the hospital ship *Albert*, off Lowestoft. For five years now this "floating bazaar" has been held here on the healing craft belonging to the Missions to Deep Sea Fishermen.

THE Port Medical Officer had a curious case to deal with the other day at Greenwich, for the decomposed body of a large whale was washed ashore there. No owner being found, it was towed down the river, and treated "as a nuisance dangerous to health."

THE overturning of an omnibus between Rottingdean and Brighton the other day resulted in 19 persons being conveyed to the County Hospital. Two were detained as in-patients, one, the driver of the vehicle, with a fractured ankle and other injuries.

IN the month of July the water analysts reported to the water examiner of the Metropolis that out of 189 specimens taken from the London water companies one was slightly turbid, one clean but dull, and all the remainder clear, bright, and well filtered.

A BAZAAR on a large scale was inaugurated the end of August at Felixstowe, promoted in aid of the funds of the Suffolk Convalescent Home. This charity, it must be observed, is not a purely local one, as subscribers living in any part of England are entitled to send patients to it.

A SOMEWHAT lively scene appears to have taken place in Mile End Road the other day, when, it is reported, a hairdresser objected to the removal of his child, who was suffering from small-pox. The ambulance is said to have made several fruitless journeys to the house, and the matter was eventually referred to the medical officers of health.

A "MEDICINE Time Indicator," patented by J. Feaver, 83, Croydon Road, Anerley, S.E., appears likely to be useful in home nursing. It consists of a label or tablet on which numbers are printed, and a moveable pointer arranged in connection with it. This pointer can be placed opposite to the figure which indicates when the next dose of medicine is due.

ANOTHER Local Government Board inquiry has been opened at Hounslow into the scheme proposed by the Corporation of Richmond and the District Council of Heston and Isleworth for the establishment of a joint isolation hospital at Mogden, in Isleworth. The new site is opposed by the Twickenham District Council and an adjoining landowner.

A SUM of £3,300 has been realised on property left to the Middlesex Hospital under the will of the Rev. T. Nicoll. Several legacies have recently been received, and the list of donations to the institution includes a further £200 from the trustees of Mr. Smith's Kensington Charity, and one from "M. S. F." for the New Wing Fund, and several sums from "grateful patients."

SOME interesting facts with regard to the closure of schools during epidemics have been given by Dr. Thursfield, M.O.H. Salop. The pecuniary loss to the schoolmaster which the postponement of an examination entails appears to lead to the schools being either kept open or opened specially for the day of the inspector's visit, when the prevalence of infectious disease renders the assemblage of the children most undesirable.

THE presence of unqualified practitioners and the absence of water are amongst the trials of the East enders. To these may be added the prescriptions of unqualified chemists according to the evidence at a recent inquest. There would be something comic in doses of tea-leaves were it not for the fact that cheap "doctors" and cheaper "physic" are created by the demands of the poor and destitute in that outlying district.

### THE STUDENT OF MEDICINE.

### APPOINTMENTS.

**W. M. Clendinnen, M.R.C.S.Eng., L.R.C.P.Lond., appointed Medical Officer of Health, Covely Urban District. C. Templeman, M.D.Edin., appointed Medical Officer of Health for Dundee. Dr. J. Thomas, appointed Medical Officer of Health to the Lowestoft Town Council. J. Winn, L.R.C.P., L.R.C.S.I., appointed Medical Officer of Health for Clayton-le-Fylde.**

### VACANCIES.

**The following vacancies are announced this week, the amount of salary in each case being given after the name of the institution:—**

### Resident Medical Officers.

Brighton, Hove, and Preston Dispensary, Queen's Road, Brighton.—Medical Officer for the No. 6 District. Applications to the Secretary by September 9th.

City of London Hospital for Diseases of the Chest, Victoria Park, E.—House Physician. Board and residence and allowance for washing provided. Appointment for six months. Applications to the Secretary by September 12th.

Derbyshire Royal Infirmary, Derby.—Clinical Assistant; must be qualified and registered under the Medical Acts of Students of Medicine, who have only their final examination to pass. Appointment for six months. An honorarium of £10 after six months' satisfactory service will be given, and board, residence, and washing. Applications and testimonials to Walter G. Carnt, Secretary, before September 13th.

General Hospital, Nottingham.—House Physician. Appointment for two years, but eligible for re-election. Salary £100 per annum, rising £10 a year to £120. Assistant House Surgeon. Appointment for six months. Board, lodging, and washing in hospital; no salary. Applications to the Secretary for the former post by September 11th, and for the latter by September 7th.

Great Yarmouth Hospital.—House Surgeon; must be doubly qualified and able when required to give lectures for probationer nurses. Salary £90 per annum, with board and lodging. Applications and testimonials to R. F. E. Ferrier, Honorary Secretary, before September 14th.

Metropolitan Hospital, Kingsland Road, N.E.—House Physician, House Surgeon, Assistant House Physician, and Assistant House Surgeon. Appointments tenable for six months. The House Physician and House Surgeon will each receive a salary at the rate of £60 a year. Must possess a registered English medical and surgical qualification. Applications and testimonials to Charles H. Byers, Secretary, before September 9th.

Lancaster Infirmary and Dispensary.—House Surgeon; unmarried; must be doubly qualified and registered. Salary £80, with residence, board, attendance, and washing. Applications to Allan Sewart, Honorary Secretary, before September 12th.

Royal United Hospital, Bath.—House Surgeon. Candidates must be M.R.C.S.Eng., and registered. Appointment for one year. Salary £60, with board, lodging, and washing. Applications and testimonials to W. Stockwell, Secretary-Superintendent, before September 11th.

### Non-Resident Medical Officers.

City of London Hospital for Diseases of the Chest, Victoria Park, E.—Assistant Physician; must be M. or F.R.C.P.Lond. Applications to the Secretary by September 14th.

Glasgow Maternity Hospital.—Obstetric Physician and Assistant Obstetric Physician. Applications to Arthur Forbes, Secretary, 146, Buchanan Street, Glasgow, by November 8th.

Plymouth Public Dispensary.—Second Medical Officer of the Provident Department. Appointed for one year, but eligible for re-election; doubly qualified. Remuneration will be the net profits (after deduction of the expenses mentioned in the scheme). Applications to the Honorary Secretary, W. H. Prance, 7, Athenæum Terrace, Plymouth, by September 11th.

St. Bartholomew's Hospital.—Assistant Physician. Candidates must be Fellows or Members of the Royal College of Physicians, London. Applicants must attend the Court of Governors to be held on Thursday, September 26th. Applications and testimonials to W. Henry Cross, Clerk, by September 9th.

### UNIVERSITIES AND COLLEGES.

### Royal College of Surgeons in Ireland.

PRIZE LIST SUMMER SESSION, 1895.

THE BARKER ANATOMICAL PRIZES.—N. H. Alcock and C. J. Patten (equal), £26 5s. each; W. J. Sweeny, special prize, £15 15s.

THE CARMICHAEL SCHOLARSHIP.—F. J. Palmer, £15.

THE THEORY AND PRACTICE OF SURGERY.—L. M'Dowell, gold medal; G. A. Robinson, silver medal; Miss C. L. Williams, certificate.

PRACTICAL HISTOLOGY.—D. Hadden, first prize; H. Hall, second prize.

PRACTICAL CHEMISTRY.—P. A. Fraser, first prize; C. C. Meek and W. J. Anglim (equal), second prize.

PUBLIC HEALTH AND FORENSIC MEDICINE.—A. I. Eades, first prize; Miss C. L. Williams, second prize.

MATERIA MEDICA.—R. H. D. Pope, first prize; G. R. M'Donald, second prize.

PRACTICAL PHARMACY.—S. R. Godkin, first prize; M. Gavin, second prize.

BIOLOGY.—M. Gavin, first prize; W. M'Lorinan and W. J. Anglim (equal), second prize.

